# Characterization of the *Drosophila* Ortholog of the Human Usher Syndrome Type 1G Protein Sans

**DOI:** 10.1371/journal.pone.0004753

**Published:** 2009-03-09

**Authors:** Fabio Demontis, Christian Dahmann

**Affiliations:** Max Planck Institute of Molecular Cell Biology and Genetics, Dresden, Germany; Institut Pasteur, France

## Abstract

**Background:**

The Usher syndrome (USH) is the most frequent deaf-blindness hereditary disease in humans. Deafness is attributed to the disorganization of stereocilia in the inner ear. USH1, the most severe subtype, is associated with mutations in genes encoding myosin VIIa, harmonin, cadherin 23, protocadherin 15, and sans. Myosin VIIa, harmonin, cadherin 23, and protocadherin 15 physically interact in vitro and localize to stereocilia tips in vivo, indicating that they form functional complexes. Sans, in contrast, localizes to vesicle-like structures beneath the apical membrane of stereocilia-displaying hair cells. How mutations in *sans* result in deafness and blindness is not well understood. Orthologs of *myosin VIIa* and *protocadherin 15* have been identified in *Drosophila melanogaster* and their genetic analysis has identified essential roles in auditory perception and microvilli morphogenesis, respectively.

**Principal Findings:**

Here, we have identified and characterized the *Drosophila* ortholog of human *sans*. *Drosophila Sans* is expressed in tubular organs of the embryo, in lens-secreting cone cells of the adult eye, and in microvilli-displaying follicle cells during oogenesis. *Sans* mutants are viable, fertile, and mutant follicle cells appear to form microvilli, indicating that Sans is dispensable for fly development and microvilli morphogenesis in the follicle epithelium. In follicle cells, Sans protein localizes, similar to its vertebrate ortholog, to intracellular punctate structures, which we have identified as early endosomes associated with the syntaxin Avalanche.

**Conclusions:**

Our work is consistent with an evolutionary conserved function of Sans in vesicle trafficking. Furthermore it provides a significant basis for further understanding of the role of this Usher syndrome ortholog in development and disease.

## Introduction

The analysis of orthologs of human disease genes in model organisms has revealed important insights into developmental mechanisms. One outstanding example is the contribution that the analysis of Usher syndrome-related genes has made to our understanding of hair cell development and auditory perception [1]. Usher syndrome is the most common hereditary disease associated with deafness and blindness in humans [2], [3], [4]. Three clinical subtypes, USH1, USH2, and USH3, have been defined according to the severity of the hearing impairment, the absence or presence of vestibular dysfunctions, and the age of onset of retinitis pigmentosa, which leads to blindness. USH1 is the most severe subtype and USH1 patients suffer from severe hearing impairment, balance defects, and prepubertal-onset of retinitis pigmentosa. Five *USH1* genes have been identified: *USH1B* encodes an unconventional motor protein, myosin VIIa [5]; *USH1C* encodes a PDZ-domain-containing scaffolding protein, harmonin [5]; *USH1D* and *USH1F* encode two members of the cadherin family of Ca^2+^-dependent cell adhesion molecules, cadherin 23 and protocadherin 15, respectively [6], [7], [8], [9]; *USH1G* encodes a putative scaffolding protein, sans, containing three ankyrin repeats and a sterile alpha motif (SAM) domain [10].

Hair cells of the mammalian inner ear display bundles of actin-filled, microvilli-derived projections of the apical membrane, known as stereocilia, that act as mechanosensitive devices important for the detection of sound [1]. Stereocilia develop from microvilli through the lateral addition of actin filaments to the microvillar actin bundle and the subsequent elongation of the filaments within the actin bundle [11]. The differential elongation of stereocilia leads to their characteristic staircase-like arrangement. Within each bundle, the stereocilia are connected by various links, including tip links connecting the tip of one stereocilium to the side of an adjacent stereocilium. Mutations in the five known *USH1* genes lead to alterations in stereocilia length and orientation. Biochemical analysis further shows that the five USH1 proteins can interact with each other in vitro. Harmonin binds, through its PDZ domain, to any of the other four USH1 proteins [10], [12], [13], [14], [15] and myosin VIIa can interact with protocadherin 15 and sans [12], [16]. Localization studies have revealed that four of the five USH1 proteins are present on stereocilia tips: myosin VIIa, cadherin 23, protocadherin 15, and harmonin [13], [17], [18]. The in vitro binding and in situ co-localization of these four USH1 proteins suggest that they form functional complexes. Cadherin 23 and protocadherin 15, furthermore, have been recently shown to be part of the tip links that connect stereocilia, thereby providing cohesion between adjacent stereocilia [19], [20]. Sans, in contrast to the other identified USH1 proteins, localizes to vesicular structures beneath the apical plasma membrane of hair cells [12] and has not been reported to reside on stereocilia. Based on its localization and physical interaction with myosin VIIa and harmonin, it has been proposed that sans may control the trafficking of USH1 proteins to stereocilia [12].

Animal models have been important in further understanding the etiology of Usher syndrome. Mice mutant for *myosin VIIa* (shaker-1), *harmonin* (deaf circler), *cadherin 23* (waltzer), *protocadherin 15* (Ames waltzer), or *sans* (Jackson shaker) display severe hearing impairment, vestibular dysfunction, and disorganized stereocilia in the inner ear [7], [18], [21], [22], [23], [24], [25]. Localization studies in mutant mice have shown that both myosin VIIa and sans are required to direct harmonin to the stereocilia tips [18]. Mutations in *myosin VIIa*, *cadherin 23*, and *protocadherin 15a* are also associated with disorganized hair bundles in zebrafish [26], [27], [28]. In *Drosophila melanogaster*, orthologs of *USH1* genes have been identified. Mutations in *crinkled*, the fly ortholog of *myosin VIIa* [29], result in disorganization of Johnston's organ, the flies' auditory system, and in deafness [30], [31]. Moreover, Cad99C, the ortholog of protocadherin 15, localizes to microvilli of the fly follicular epithelium, and mutations in this cadherin result in the misorientation of microvilli [32], [33]. These findings indicate an evolutionary conserved function for this class of cadherins in organizing actin-containing cellular protrusions.

Here, in order to further understand the role of *sans* during development and disease, we identified and characterized a *Drosophila melanogaster* ortholog of this gene. We show that *Drosophila Sans* is expressed in tubular organs in the embryo, lens-secreting cone cells in the adult eye, and microvilli-displaying follicle cells during oogenesis. Similar to its vertebrate ortholog, Sans protein localizes to punctate structures we identified as early endosomes associated with the syntaxin Avalanche.

## Results

### The *Drosophila* genome encodes an ortholog of the human Usher syndrome type 1G protein sans

**Figure 1:**
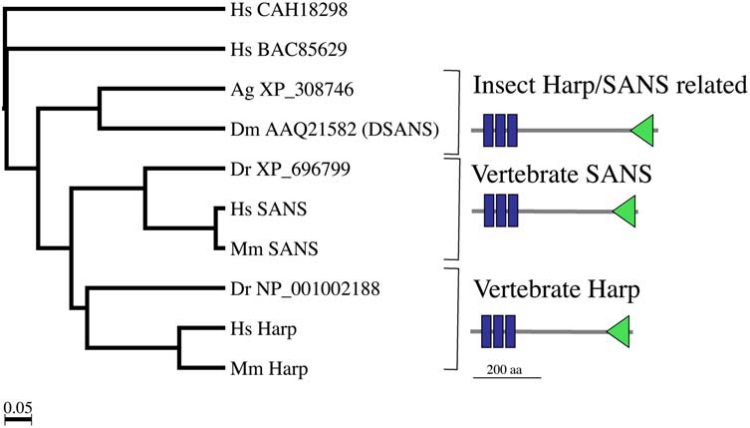
Identification of a *Drosophila melanogaster* ortholog of human Usher syndrome type 1G protein sans. Phylogenetic tree. *Drosophila melanogaster* Sans is closely related to vertebrate sans/Harp proteins. Harp/sans proteins contain three ankyrin repeats (blue rectangles) and one SAM domain (green triangle). Hs: *Homo sapiens*; Ag: *Anopheles gambiae*; Dm *Drosophila melanogaster*; Dr: *Danio rerio*; Mm: *Mus musculus*.

To identify the *Drosophila* ortholog of the human sans protein, we used the human sans protein sequence in a BLASTp search against the *Drosophila melanogaster* genome. The product encoded by gene *CG13320* was most similar to human sans. Back-Blast using CG13320 protein as a query sequence retrieved sans as the most related protein in vertebrates. Overall, human sans and *Drosophila* CG13320 are 32% identical (168/524 identical amino acid residues). A higher sequence identity was detected in the N-terminus (53%; 15/28), the ankyrin repeats (60%, 53/88), and the SAM domain (39% (26/66). Human sans and *Drosophila* CG13320 share a conserved C-terminal PDZ-domain binding site (data not shown) and display a similar domain organization including three ankyrin repeats and one SAM domain (Fig. 1). Moreover, in a phylogenetic tree, CG13320 clustered in the same clade as vertebrate sans and harp (Fig. 1). Harp is an ankyrin and SAM-domain containing protein related to sans. We conclude that *CG13320* is the *Drosophila* ortholog of human *sans* and refer to it as *Sans* (see also FlyBase at www.flybase.org).

### *Sans* is expressed in tubular organs of the embryo and in ovarian follicle cells

**Figure 2:**
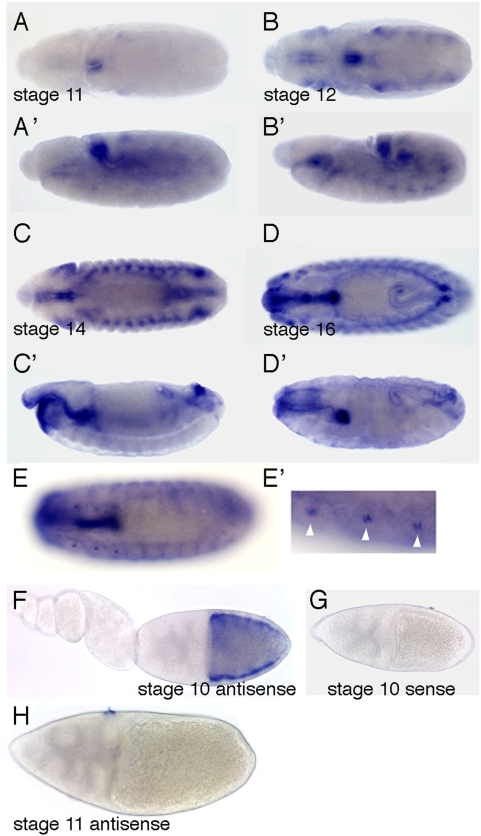
*Sans* is expressed in tubular organs of the embryo and in ovarian follicle cells. A–E′: Wild-type embryos of the indicated stages hybridized with a *Sans*-specific antisense RNA probe. A,B,C,D,E: Dorsal views. A′,B′,C′,D′: Lateral views. E′ is a magnification of E showing the Keilin's organs (arrowheads). F–G: Wild-type ovaries of the indicated stages hybridized with (F,H) a *Sans*-specific antisense RNA probe or (G) a sense RNA probe. Sans is expressed in follicle cells surrounding the oocyte in stage 10 egg chambers. Anterior is to the left.

To further characterize *Drosophila Sans*, we analyzed its expression pattern in embryonic, larval, and adult tissues by RNA in situ hybridization using a *Sans*-specific RNA probe. In the embryo, *Sans* mRNA was first detectable at stage 11 in the hindgut (Fig. 2A). In later stages, *Sans* mRNA was detected in the foregut, the hindgut, the tracheal placodes, the tracheal primary branches, and the six mechanosensory Keilin's organs (Fig. 2B–E′). During larval stages, *Sans* was expressed ubiquitously in eye-antennal, wing, leg, and haltere imaginal discs (data not shown). The adult *Drosophila* ovary is composed of chains of egg chambers proceeding through 14 stages from the germarium to the oviduct [34]. Each egg chamber consists of 16 germline cells, one oocyte and 15 nurse cells, encapsulated by a monolayer of somatic, epithelial follicle cells. *Sans* expression was confined to follicle cells contacting the oocyte in stage 9–10A egg chambers (Fig. 2F,H), a time when follicle cell microvilli are remodeled and elongate. *Sans* RNA localized to the apical side of follicle cells (Fig. 2F). No staining was detected using a sense RNA probe to *Sans* (Fig. 2G), indicating that the staining was specific.

### *Sans* is dispensable for fly development and fertility

**Figure 3:**
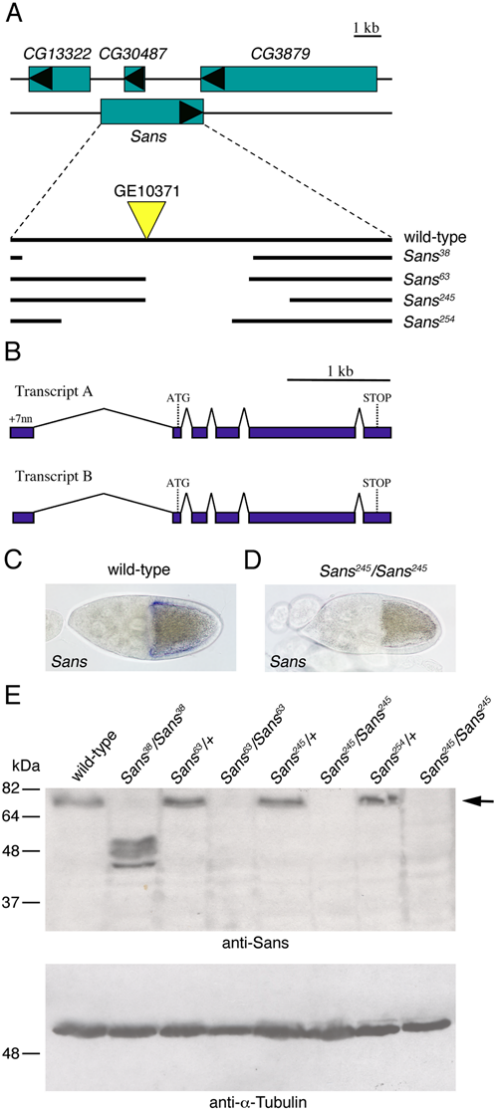
Genomic organization of *Sans* and characterization of *Sans* mutant alleles. A: Genomic organization in the region of *Sans* and extent of the deletions in *Sans* mutant alleles. The position of the EP-element *GE10371* which was used to generate the *Sans* mutants is indicated. B: Exon-intron structure of *Sans*. The scale and genomic position is the same as for the extent of the deletions shown in (A). The two *Sans* transcripts differ by an addition of seven nucleotides to the 5′ end of transcript A. C,D: Stage 10 (C) wild-type and (D) *Sans^245^/Sans^245^* mutant egg chambers hybridized with a *Sans*-specific RNA probe. The hybridization signal is undetectable in the mutant. E: Western blot analysis of ovaries with an anti-Sans antiserum. Genotypes of the flies are indicated. The anti-Sans antiserum detects a protein of ∼75 kDa (arrow), the expected molecular weight for Sans, in extracts of wild-type (lane 1) and heterozygous (lanes 3,5,7), but not homozygous (lanes 2,4,6,8) *Sans* mutant ovaries. Subsequent blotting with an anti α-Tubulin antibody (lower panel) shows the loading of similar amounts of protein in each lane. Molecular weights are indicated to the left (in kDa).

To functionally analyze *Sans*, we generated four different mutant alleles of *Sans* by imprecise excision of EP-element *GE10371* inserted in the first intron of the *Sans* gene (Fig. 3A,B). All four mutations deleted parts of the predicted coding sequence for *Sans*, but did not obviously alter neighboring genes. These mutations, however, affect the putative gene *CG30487*, whose predicted coding sequence lies on the complementary DNA strand within an intron of *Sans*. Analysis by in situ hybridization with a *Sans* specific RNA probe showed that *Sans* transcript could no longer be detected in *Sans^245^/Sans^245^* mutant egg chambers (Fig. 3C,D), suggesting that expression of *Sans* was impaired from this mutant allele. To test this further, we performed Western-blot analysis of control and *Sans* mutant ovaries using a polyclonal anti-Sans antiserum raised against the full-length protein (see Materials and Methods). A protein of approximately 75 kDa was detected in wild-type or control heterozygous mutant ovaries (Fig. 3E). Proteins of approximately 50 kDa, possibly corresponding to truncate forms of Sans, were detected in *Sans^38^/Sans^38^* mutant flies, suggesting that this allele is hypomorphic (Fig. 3E). However, no Sans protein was detected in *Sans^63^/Sans^63^*, *Sans^245^/Sans^245^*, and *Sans^254^/Sans^254^* homozygous mutant flies (Fig. 3E), suggesting that these alleles are amorphic. Flies homozygous mutant for any of the four *Sans* alleles were adult viable, male and female fertile, and had no gross morphological defects (data not shown), indicating that Sans is not essential for fly development and fertility.

### Sans, Cad99C, and Crinkled/Myosin VIIa localize to cone cells in the adult eye

**Figure 4:**
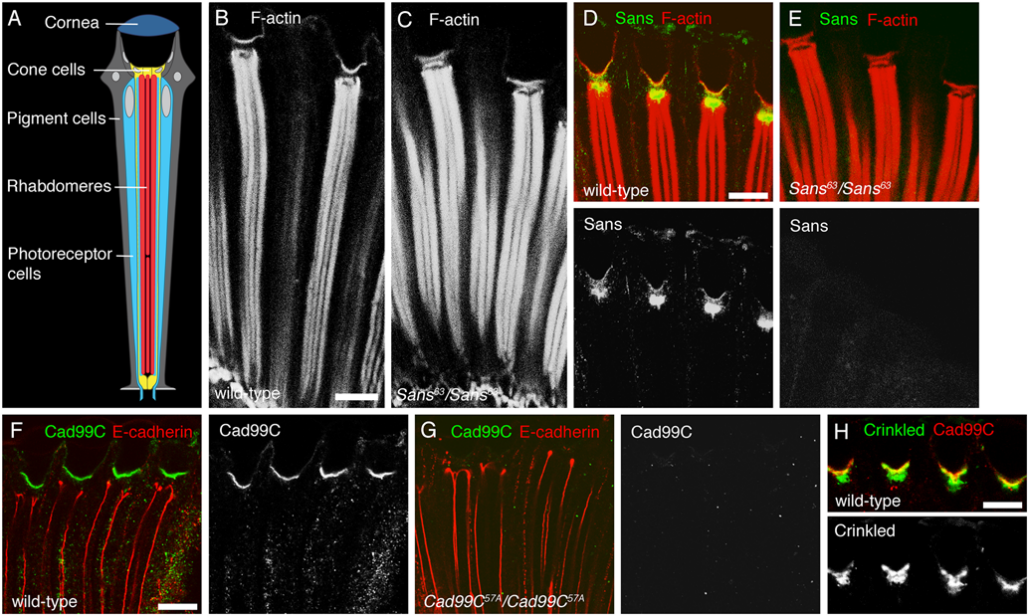
Localization of Sans, Cad99C, and Crinkled proteins in adult retinae. A: A scheme of a longitudinal section of a *Drosophila* adult ommatidium. B–C: Longitudinal optical sections of adult retinae from (B) wild-type and (C) *Sans^63^/Sans^63^* mutant animals stained for Rhodamine-Phalloidin (red) to detect Filamentous (F-) actin. D–E: Longitudinal optical sections of adult retinae from (D) wild-type and (E) *Sans^63^/Sans^63^* mutant animals stained for Sans (green) and F-actin (red). F–G: Longitudinal optical sections of adult retinae from (F) wild-type and (G) *Cad99C^57A^/Cad99C^57A^* mutant animals stained for Cad99C (green) and E-cadherin (red). E-cadherin labels the adherens junctions between photoreceptor cells. H: Longitudinal optical sections of wild-type adult retinae stained for Crinkled (green) and Cad99C (red). Sans, Cad99C, and Crinkled localize to cone cells. Scale bars: 10 µm.

In the vertebrate eye, a number of USH1 proteins have been localized to the ciliary and periciliary regions of photoreceptor cells (reviewed in [35]). In addition, myosin VIIa is expressed in the retina pigmented epithelium [36], a multifunctional epithelium placed between the photoreceptors and the vascular meshwork of the choroid layer. To determine the localization of the three USH1 orthologs Sans, Cad99C, and Crinkled/Myosin VIIa in the *Drosophila* eye, we stained adult eyes with antisera specific to these proteins. The adult compound eye is a regular hexagonal array of approximately 750 ommatidia. Each ommatidium consists of 8 photoreceptors, 4 cone cells, and pigment cells (Fig. 4A). The cone cells overlie the photoreceptors and secrete lens material from their apical side. Photoreceptors display rod-like stacks of photosensitive microvilli at their apical surfaces, called rhabdomeres. The overall morphology of rhabdomeres, as visualized by staining for F-actin, was indistinguishable between control and *Sans^63^/Sans^63^* mutant flies (Fig. 4B,C). Sans immunoreactivity was strongly detected in cone cells (Fig. 4D). No immunoreactivity was detected in the adult eyes of *Sans^63^/Sans^63^* mutant flies (Fig. 4E), indicating that the observed staining was specific to Sans. In addition, we detected Cad99C immunoreactivity at what appeared to be the apical side of cone cells in control, but not in *Cad99C^57A^/Cad99C^57A^* mutant flies (Fig. 4F,G). Finally, Crinkled/Myosin VIIa immunoreactivity was detected in cone cells in control, but not in *ck^13^/ck^13^* mutant flies (Fig. 4H and data not shown). We conclude that Sans, Cad99C, and Crinkled/Myosin VIIa localize to cone cells in adult eyes.

### Sans, Cad99C, and Crinkled/Myosin VIIa localize to follicle cells

**Figure 5:**
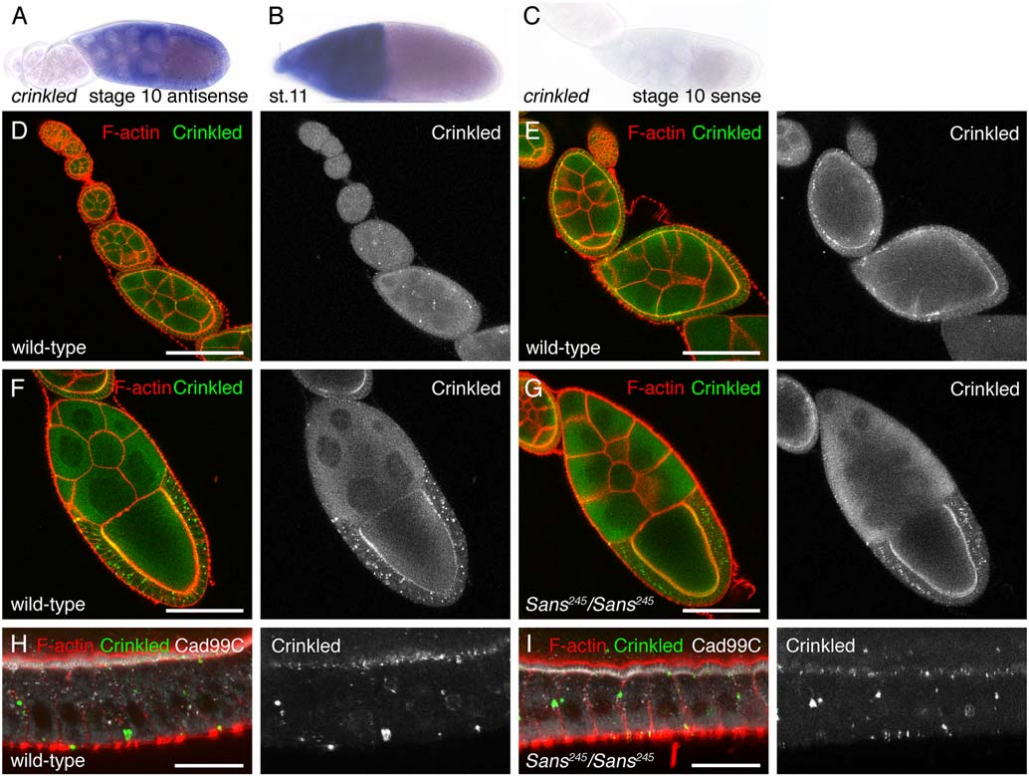
Expression and localization of Crinkled/Myosin VIIa in ovaries. A–C: Wild-type ovaries of the indicated stages hybridized with (A,B) a *crinkled*-specific antisense RNA probe or (C) a sense RNA probe. *crinkled* is expressed in follicle cells surrounding the oocyte in stage 10 egg chambers. D–G: Ovaries of (D–F) wild-type and (G) *Sans^245^/Sans^245^* mutant flies stained for Crinkled/Myosin VIIa (green) and F-actin (red). In (F,G) stage 10 egg chambers are shown. H,I: High magnification views of follicle cells of stage 10 egg chambers of (H) wild-type and (I) *Sans^245^/Sans^245^* mutant flies stained for Crinkled/Myosin VIIa (green), F-actin (red) and Cad99C (white). Crinkled/Myosin VIIa localizes to punctate structures at the apical side of follicle cells and, in part, to Cad99C-stained microvilli in wild-type and *Sans^245^/Sans^245^* mutant flies. Scale bars: D–G: 100 µm; H,I: 20 µm.

**Figure 6:**
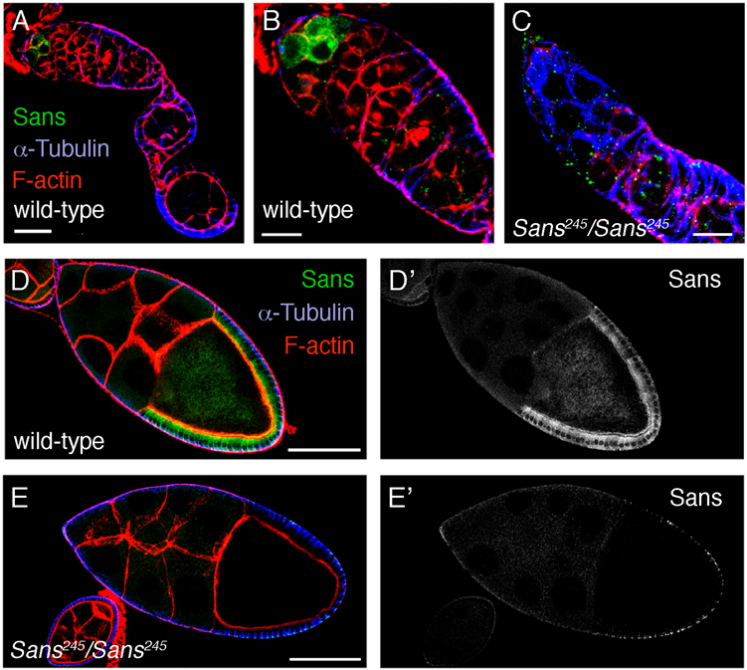
Localization of Sans protein in ovaries. A–E: Ovaries of (A,B,D) wild-type and (C,E) *Sans^245^/Sans^245^* mutant flies stained for Sans (green), α-Tubulin (blue), and F-actin (red). (A–C) show the germarium and (D,E) stage 10 egg chambers. In (D′,E′) only the Sans staining is shown. Sans is expressed in what appears to be the germline stem cells of the germarium (A,B) and in follicle cells contacting the oocyte (D). Sans staining is also detected at the cortex of the oocyte. Anterior is to the left. Scale bars: A: 50 µm; B,C: 10 µm; D,E: 100 µm.

Cad99C has previously been shown to localize to microvilli of follicle cells in stage 10 egg chambers [32], [33]. We next determined the expression and localization of Crinkled/Myosin VIIa and Sans in ovaries. *crinkled* RNA was detected in nurse cells and follicle cells of stage 9–10 egg chambers (Fig. 5A). In stage 11, *crinkled* RNA was confined to the nurse cells (Fig. 5B). No staining was detected using a sense RNA probe to *crinkled* (Fig. 5C), indicating that the staining was specific. Crinkled/Myosin VIIa immunoreactivity was first detected during oogenesis at the apical side of follicle cells in stage 7 egg chambers (Fig. 5D,E). In stage 10 egg chambers, immunoreactivity was detected in the follicle cells surrounding the oocyte and in the germline (Fig. 5F), consistent with the expression of *crinkled* RNA. Similar to *crinkled* RNA, Crinkled/Myosin VIIa immunoreactivity was no longer detected in follicle cells of stage 11 egg chambers (data not shown). Higher magnification views of stage 10 egg chambers showed that Crinkled/Myosin VIIa immunoreactivity was present in punctate structures at the apical side of follicle cells and, in part, co-localized with Cad99C (Fig. 5H). Crinkled/Myosin VIIa immunoreactivity was not obviously altered in ovaries of *Sans^245^/Sans^245^* mutant flies (Fig. 5G,I). Sans immunoreactivity was detected in the most anterior part of the germarium in what appeared to be the germline stem cells (Fig. 6A,B). Moreover, Sans immunoreactivity was observed in the follicular epithelium surrounding the oocyte of control stage 9–10A egg chambers (Fig. 6D,D′), consistent with the pattern of *Sans* mRNA expression. No immunoreactivity was detected in ovarioles of *Sans^245^/Sans^245^* mutant flies (Fig. 6C, E,E′), indicating that the observed stainings were specific for Sans. We conclude that Sans, Cad99C, and Crinkled/Myosin VIIa localize to follicle cells.

### Sans co-localizes with the syntaxin Avalanche to early endocytic vesicles in follicle cells

**Figure 7:**
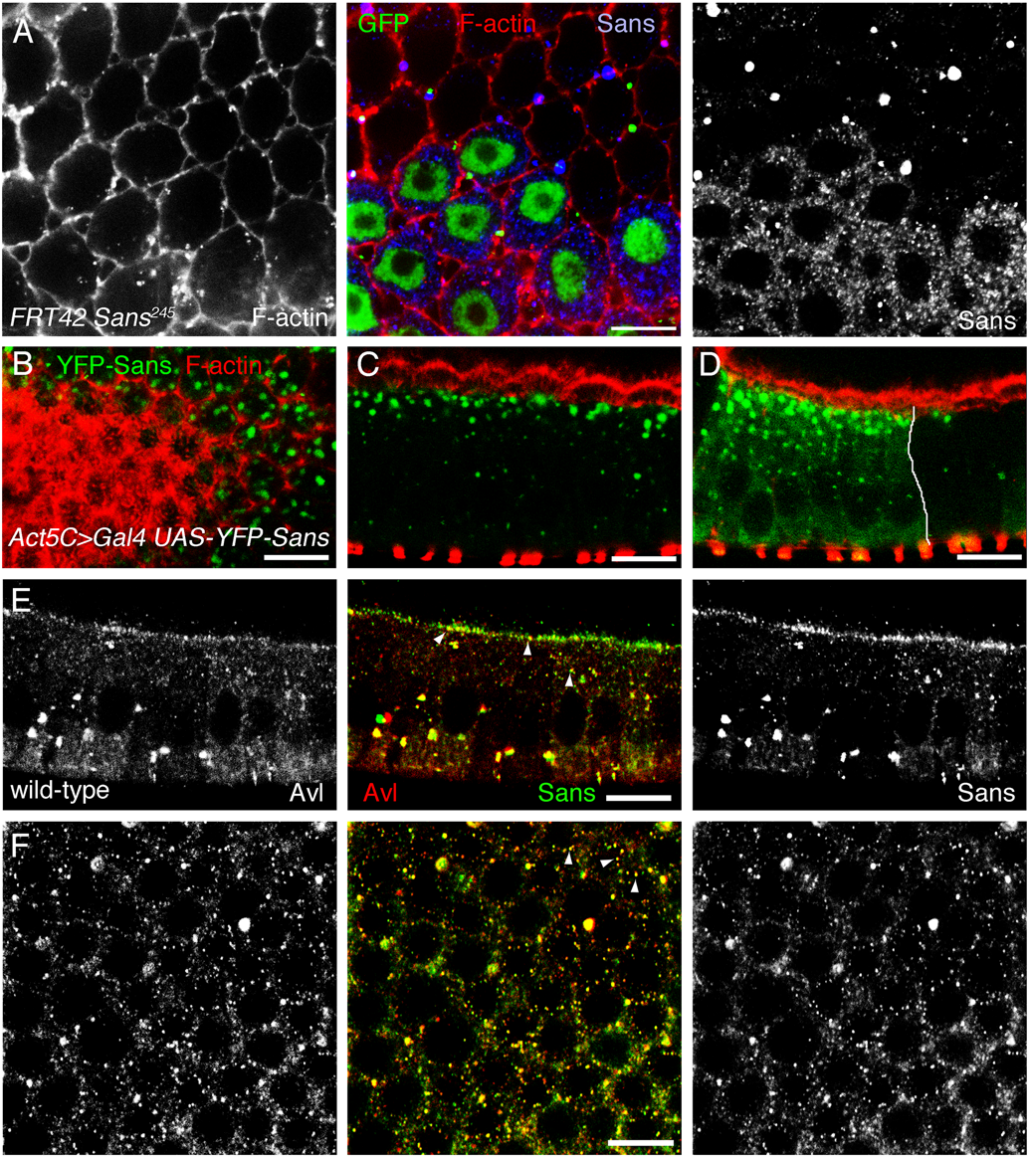
Sans co-localizes with the syntaxin Avalanche to early endocytic vesicles. A: Clones of *Sans^245^/Sans^245^* mutant follicle cells marked by the absence of nuclear GFP (green) of stage 10-egg chambers stained for F-actin (red) and Sans (blue). Sans localizes to intracellular punctate structures of follicle cells. B–D: Clones of follicle cells expressing YFP-Sans (*Act5C>Gal4*, *UAS-YFP-Sans*) labeled by YFP-Sans (green) and stained for F-actin (red). The white line in (D) delineates the clone border. YFP-Sans localizes to punctate structures enriched beneath the apical plasma membrane. Note that the YFP-Sans punctate structures are enlarged in comparison to the endogeneous Sans punctate structures. E,F: Wild-type stage 10-egg chambers stained for Avalanche (Avl, red) and Sans (green). Sans partially co-localizes with Avl in punctate structures. (A,F) show top views and (B–E) show cross-sectional views of the follicle epithelium (apical is to the top). Scale bars: A,C–F: 10 µm; B: 20 µm.

Next we analyzed in more detail the subcellular localization of Sans in follicle cells. Higher magnification views revealed that Sans localized to punctate structures within the follicle cell cytoplasm (Fig. 7A). Similarly, a YFP-Sans fusion protein, expressed using the GAL4-UAS-system, localized to intracellular punctate structures (Fig. 7B). Sans, as well as the YFP-Sans fusion protein, were enriched beneath the apical membrane of follicle cells (Fig. 7C–E).

The syntaxin Avalanche (Avl) has been previously shown to localize in follicle cells to punctate structures within the cytoplasm below the apical plasma membrane [37], similar to Sans. Avl is present in an early compartment of the endocytic pathway and is required for endocytosis of the Notch and Crumbs transmembrane proteins [37]. To test whether Sans and Avl localize to the same punctate structures, we stained follicle cells for Sans and Avl. Sans-labelled punctate structures frequently co-localized with Avl (Fig. 7E,F). Image analysis revealed that 78% (n = 319) of the Sans-labelled punctate structures were also co-labeled by the Avl antibody. We conclude that Sans, at least in part, localizes to Avl-associated early-endocytic vesicles.

### Sans is apparently not required for the normal morphogenesis of follicle cell microvilli

**Figure 8:**
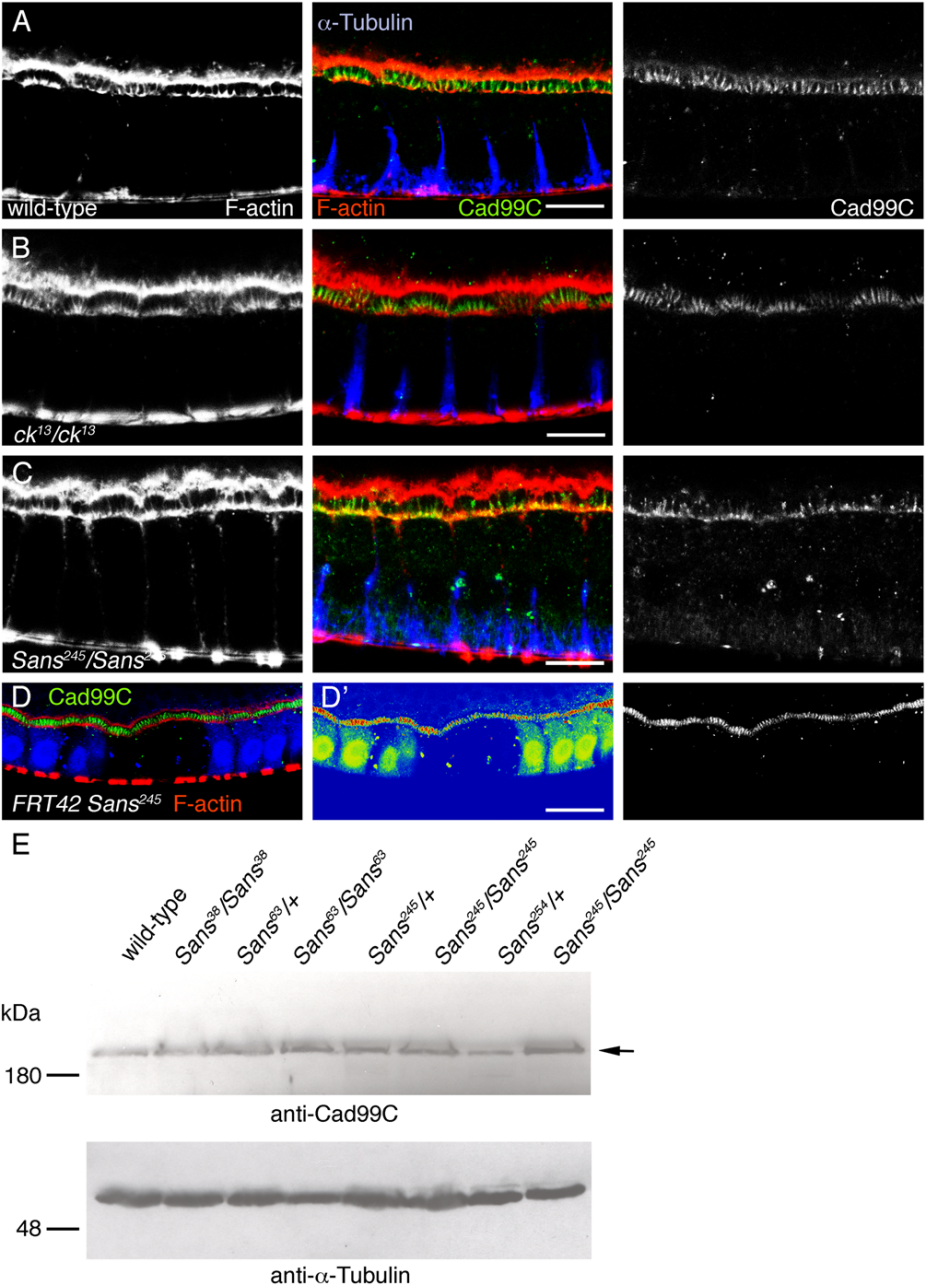
Follicle cell microvilli display an apparently normal morphology in *Sans* and *ck* mutant flies. A–C: Cross-sectional views of (A) wild-type, (B) *ck^13^/ck^13^* mutant, and (C) *Sans^245^/Sans^245^* mutant follicle cells of stage 10-egg chambers stained for F-actin (red), Cad99C (green), and α-Tubulin (blue). At this level of resolution, microvilli appear to be normal in the mutants. D: Clones of *Sans^245^/Sans^245^* mutant follicle cells marked by the absence of GFP (blue) of stage 10-egg chambers stained for F-actin (red) and Cad99C (green). A cross-sectional view is shown (apical is to the top). D′ displays a heat-map of the staining shown in D. Cad99C is reduced on microvilli of *Sans^245^/Sans^245^* mutant follicle cells. E: Western blot analysis of ovaries with an anti-Cad99C antiserum. Genotypes of the flies are indicated. Similar amounts of Cad99C (arrow) are detected in extracts of wild-type (lane 1), heterozygous (lanes 3,5,7), and homozygous (lanes 2,4,6,8) *Sans* mutant ovaries. Subsequent blotting with an anti α-Tubulin antibody (lower panel) shows the loading of similar amounts of proteins in each lane. Molecular weights are indicated to the left (in kDa). Scale bars: A–C: 10 µm; D: 20 µm.

Mutations in *Cad99C*, the ortholog of the human Usher syndrome type 1F protein protocadherin 15, result in an abnormal morphology of follicle cell microvilli [32], [33]. Similar to *Cad99C*, *Sans* is expressed in stage 9–10A follicle cells, prompting us to test whether Sans is also required for normal microvilli morphogenesis. To this end, we immunostained stage 10 egg chambers of control and *Sans^245^/Sans^245^* mutant flies for F-actin, which identifies microvilli. As shown in Fig. 8 (A and C), the morphology of follicle cell microvilli, as revealed by this analysis, was indistinguishable between control and *Sans^245^/Sans^245^* mutant flies. Moreover, follicle cell microvilli also appeared to be normal in mutants for *crinkled (ck^13^)/myosin VIIA* (Fig. 8B). Cad99C, a marker for microvilli [32], [33], appeared to localize normally on microvilli of *ck^13^/ck^13^* mutant follicle cells, as analyzed by immunofluorescence (Fig. 8A,B). Cad99C also localized to microvilli in *Sans^245^/Sans^245^* mutant follicle cells, even though Cad99C staining on microvilli appeared, in some instances, to be less pronounced compared to controls (Fig. 8C,D). The total amount of Cad99C in ovaries, as analyzed by Western blotting, was similar in control and *Sans* mutant flies (Fig. 8E). Thus, unlike Cad99C, Sans does not appear to play an essential role during microvilli morphogenesis in the follicle epithelium.

## Discussion

In this study, we have identified and characterized a *Drosophila* ortholog of the Usher syndrome protein sans. *Drosophila* Sans is expressed in tubular organs of the embryo and in cone cells in the adult eye. In the ovary, Sans is expressed in germline stem cells and in follicle cells during stages 9–10A. In follicle cells, Sans colocalizes with Avalanche, a syntaxin associated with early endosomes.

### Orthologs of *USH1* genes in *Drosophila*

The *Drosophila melanogaster* genome encodes genes orthologous to the five known *USH1* genes: *crinkled/myosin VIIa* (*USH1B*) [29], *CG5921* (*USH1C*) (data not shown), *Cad88C* (USH1D) [38], *Cad99C* (*USH1F*) [32], [33], and *Sans* (*USH1G*) (this study), providing an opportunity to study the cellular and developmental roles of these genes in this genetically tractable model organism. The analysis of *crinkled* has revealed a striking parallel in the role of this gene and *USH1B* for auditory perception. *crinkled* mutants fail to properly develop the flies auditory organs and they are deaf [30], [31]. Measurements of auditory mechanics and nerve responses indicate that *Sans* mutants do not have auditory defects (Martin Göpfert, personal communication). A further cell type in which orthologs of *USH1* genes have been analyzed in *Drosophila* is the follicle epithelium [32], [33]. Like other epithelia, the follicle cells display microvilli at their apical surface. Follicle cell microvilli are dynamic in that they extend in length during stages 9–10A of oogenesis and that they disappear during the later stages. Interestingly, all five Usher syndrome type 1 orthologs, *Cad99C*, *Cad88C*, *CG5921*, *crinkled*, and *Sans*, are expressed in follicle cells when they display long microvilli [32], [33], [39] (this study). At least three Usher syndrome type 1 proteins share similar subcellular localizations in *Drosophila* follicle cells and vertebrate hair cells. Cad99C/protocadherin 15 and Crinkled/myosin VIIa localize to microvilli/stereocilia. Sans localizes to vesicles beneath the apical surface of cells. Moreover, in the adult *Drosophila* eye, Sans, Cad99C, and Crinkled/Myosin VIIa localize to a single cell type, the lens-secreting cone cell. Based on their localization, we speculate that Sans, Cad99C, and Crinkled/Myosin VIIa might be part of a protein network, similar to what has been proposed for their vertebrate orthologs [12], [16].

### Sans and microvilli morphogenesis

Mutations in *Cad99C* result in misorientation of follicle cell microvilli [32], [33]. Normal microvilli orientation appears physiologically important, since misorientation of microvilli in *Cad99C* mutants correlates with an impairment of eggshell deposition necessary to protect the egg and developing embryo from its environment [32], [33]. In contrast to *Cad99C* mutants, follicle cell microvilli appear to be normal in both *Sans* and *crinkled* mutants, although we cannot exclude subtle defects. *Sans* mutant flies also deposit eggs surrounded by an apparently integer eggshell, as revealed by the exclusion of the dye neutral red (data not shown). In contrast to the vertebrate hair cells, microvilli morphogenesis in the follicle epithelium appears to be more robust towards single gene perturbations. We find that, although single mutants for *Sans* or *crinkled* are adult viable [29] (this study), animals double mutant for these genes do not develop to adulthood (data not shown), underscoring the developmental role of Usher syndrome orthologs in *Drosophila*. Future work will be needed to analyze microvillar morphogenesis in cells double mutant for *Sans* and *crinkled* and to determine the roles of the two thus far uncharacterized Usher type 1 orthologs *Cad88C* and *CG5921* during *Drosophila* development.

### A role for Sans in vesicle trafficking?

Based on the vesicle-like localization of sans in mammalian hair cells, it has been speculated that sans might be involved in vesicle trafficking and, in particular, the trafficking of cadherin 23 or protocadherin 15 to stereocilia [12]. However, the identity of the vesicle to which sans associated had not been revealed. We now show that, in *Drosophila* follicle cells, Sans to a large extent co-localizes with the syntaxin Avalanche to endocytic vesicles beneath the apical plasma membrane. These results are consistent with an evolutionary conserved role of Sans in vesicle trafficking. The decreased amount of Cad99C on microvilli, which we detected with low penetrance in *Sans^245^/Sans^245^* mutant follicle cells is, furthermore, suggestive of a function of Sans in the trafficking of Cad99C. Our work provides a basis for the further analysis of Usher syndrome-related genes in *Drosophila*.

## Materials and Methods

### Bioinformatic analysis

BLAST searches [40] were performed against the non-redundant protein National Center for Biotechnology Information (NCBI) database. The Simple Modular Architecture Research Tool (SMART) [41] was used to predict the domain organization of sans-related molecules across evolution. Phylogenetic analysis was performed as previously described [42]. Accession numbers of the sequences used in this study are: Hs BAC85629, Hs CAH18298, Hs SANS (NP_775748), Mm SANS (NP_789817), Hs Harp (NP_665872), Mm Harp (NP_082361) [43], Dm AAQ21582 (DSANS), Ag XP_308746, Dr NP_001002188, Dr XP_696799.

### Molecular cloning

To clone the *CG13320/Sans* coding sequence together with its 5′ and 3′ untranslated regions, we first isolated total RNA from third instar *y w* larvae using the RNeasy mini kit (Qiagen). cDNA was synthesized with the SMART RACE cDNA Amplification kit (Clontech) and used as template in a PCR reaction using the primers 5′-CCGGAATTCGCTTCCGGGTAACGGCTTTCGGAACG-3′ and 5′-CGGGGTACCGGAGTGATAATTGTTAGGCTTTAATTTAG-3′ (the underlined sequences are *EcoR*I and *Kpn*I restriction sites used for cloning), corresponding to nucleotides 1–27 and 1833–1861 of the EST sequence LD20463. The sequencing of distinct PCR products revealed the presence of two different isoforms of *CG13320* transcripts, one corresponding to the LD20463 sequence (+7nn isoform) and the other lacking 7 nucleotides in the 5′ untranslated region (nucleotides 163–169 of LD20463, −7nn isoform). The translation initiation site was identified at position 249 in the sequence NM_176160 (position 216 in the sequence LD20463) on the basis of the presence of a Kozak consensus sequence (cgaatATG), partially corresponding to the consensus sequence previously reported [44]. The translation initiation site was preceded by Stop codons in all three reading frames. The *CG13320* gene encodes a protein of 516 amino acids that differs at positions 405 (T405S) from the sequence deposited for CG13320 (AAQ21582), probably due to polymorphisms between the *Drosophila* strains used for genome sequencing and the *y w* strain used in this study.

To generate the *UAS-YFP-Sans* transgene, the *CG13320* coding sequence, together with the 3′ untranslated region, was amplified by PCR using the primers 5′-CCGGAATTCATGTCATCGGATCGG TTTCACAAAGC-3′ and 5′-CGGGGTACCGGAGTGATAATTGTTAGGCTTT AATTTAG-3′ (the underlined sequences are *EcoR*I and *Kpn*I restriction sites used for cloning), corresponding to nucleotides 216–241 and 1833–1861 of LD20463. The resulting PCR product was cloned in the pUAST vector [45]. Enhanced yellow fluorescent protein (EYFP) was PCR amplified from pEYFP-N1 (Clontech), using the primers 5′-CCGGAATTCGTCGCCACCATGGTGAGCAA GG-3′ and 5′-CCGGAATTCCTTGTACAGCTCGTCCATGCC-3′ (the underlined sequences are *EcoR*I restriction sites used for cloning). The resulting PCR product was cloned 5′ to and in frame with the *Sans* coding sequence in the pUAST vector. The correct nucleotide sequence of the cloned PCR products were confirmed by sequencing prior to injection into *y w* embryos to obtain transgenic flies.

### Fly stocks

Mutant alleles for *CG13320* were generated by imprecise excision of EP-elements [46] using standard procedures. The starting EP-element line was *GE10371* (GenExel, Inc.), in which the EP-element is oriented with the UAS sites facing away from the *CG13320* coding sequence. The deficiency line used was *Df(2R)CX1*, *wg^12^ b^1^ pr^1^/SM1* (Bloomington Drosophila stock center at Indiana University). The allele *Sans^38^* contains a genomic deletion of 2135 bp, spanning from nucleotide 121224 to 123357 of the genomic sequence AE003820. The allele *Sans^63^* contains a genomic deletion of 965 bp from nucleotide 122363 to nucleotide 123328. The allele *Sans^245^* contains a genomic deletion of 1331 bp, spanning from nucleotide 122367 to nucleotide 123696. The allele *Sans^254^* contains a genomic deletion of 1593 bp, spanning from nucleotide 121561 to nucleotide 123152. The four *Sans* alleles described here are homozygous viable, indicating that *CG13320* (*Sans*) is not essential for viability. This conclusion is consistent with previous independently conducted genetic studies which did not uncover any vital gene between *CG3845* (*l(2)01424*) and *CG3886* (*Psc*) [47], [48], an interval including *Sans*. Additional fly stocks used were *FRT42D Sans^245^* (this study), *UAS-YFP-Sans* (this study), *Act5C>CD2>Gal4* [49], and *ck^13^* [29]. Marked clones of mutant cells were generated by Flp-mediated mitotic recombination subjecting flies once to a 35°C heat-shock for 30 minutes (*Act5C<CD2>Gal4*) or three times to a 38°C heat shock for 30 minutes (*FRT42D Sans^245^*).

### RNA in situ hybridization

RNA in situ hybridization was performed as described previously [33]. The EST LD20463 was used as a template for RNA probe preparation.

### Western blotting

Western blotting was performed as described previously [33]. Primary antibodies used were rabbit anti-Sans (1∶1000; this study), rabbit anti-Cad99C, 1∶1000 [42] and mouse anti-α-Tubulin, 1∶1000 (Sigma T9026). Secondary antibodies used were HRP-conjugated goat anti-rabbit, 1∶5000 (Santa Cruz sc-2054) and HRP-conjugated goat anti-mouse, 1∶5000 (Pierce Biotechnology 31430).

### Immunohistochemistry

Immunostaining of adult retinae was performed as described previously [50]. Ovaries were dissected and prepared for immunostaining using standard protocols. The anti-Sans antibody (AE5E) was generated by immunizing rabbits using a GST-Sans (full-length) fusion protein. The antiserum was immunopurified using a resin coupled to MBP-Sans. The anti-Sans antiserum was used at a 1∶100 dilution. Additional primary antibodies were monoclonal mouse anti-α-Tubulin, 1∶100 (Sigma T9026), monoclonal mouse anti-GFP, 1∶2000 (Clontech 8362-1), chicken anti-Avl, 1∶1000 [37], rat anti E-cadherin (DCAD2), 1∶20 [51], guinea pig anti-Crinkled, 1∶100 (Fig. 4) [29], mouse anti-MYO7A 138-1, 1∶100 (Fig. 5, Developmental Studies Hybridoma Bank), and rabbit anti-Cad99C, 1∶10.000 [42]. Secondary antibodies used were Alexa 488, Alexa 594 (Molecular Probes) or CY5 (Jackson ImmunoResearch) conjugated anti-mouse, anti-guinea pig, anti-rat, or anti-rabbit IgG, all diluted 1∶200. Rhodamine-Phalloidin, used at a 1∶200 dilution, was from Molecular Probes. Confocal images were recorded on a Zeiss LSM510 microscope.
